# Diphtheria diagnostic capacity in the Western Pacific Region

**DOI:** 10.5365/wpsar.2019.10.2.008

**Published:** 2019-12-26

**Authors:** Santosh Gurung, Amy Trindall, Lucy Reeve, Adroulla Efstratiou, Varja Grabovac

**Affiliations:** a Expanded Programme on Immunization Unit, Division of Communicable Diseases, WHO Regional Office for the Western Pacific, Manila, Philippines.; b Public Health England, London, United Kingdom.

Diphtheria is an acute infectious disease affecting the upper respiratory tract and occasionally the skin and is caused by the action of diphtheria toxin produced by *Corynebacterium diphtheriae*, *Corynebacterium ulcerans* and *Corynebacterium pseudotuberculosis*. Corynebacterium infections are usually difficult to control due to their epidemic patterns, the emergence of new strains, novel reservoirs and their dissemination to susceptible human and animal populations. (*1*) Although *C. diphtheriae* is largely controlled through mass immunization programmes, diphtheria escalated to epidemic proportions within the Russian Federation and the former Soviet Republics in the 1990s, highlighting the potential for this disease to cause morbidity and mortality when immunization programmes are disrupted. (*2*) A recent review of global diphtheria epidemiology, which included an analysis of cases and information about age, showed age distribution shifts and found that the majority of cases occur in adolescents and adults. (*3*) Shifts in age distribution, from children to adolescents and adults, were observed from countries in the Western Pacific Region such as the Lao People's Democratic Republic, (*4*) the Philippines (*3*) and Viet Nam. (*5*)

Early and accurate microbiological diagnosis of each suspected case is essential to inform management and treatment of the case and close contacts. To assess the diphtheria diagnostic capacity across laboratories in the Western Pacific Region, a survey was undertaken as part of a gap analysis (see Appendix 1) by the World Health Organization Collaborating Centre for Diphtheria and Streptococcal Infections with the WHO Regional Office for the Western Pacific. The objectives of the gap analysis were to:

assess current microbiological capability for the laboratory diagnosis of diphtheria in the Western Pacific Region;
assess public health impact in individual countries where diphtheria diagnostic activities may be limited;
assess availability of specialized reagents for diphtheria diagnostics in the Western Pacific Region;
assess training needs for scientists/medical/public health staff in this specialized area and identify best practices/gaps in diphtheria diagnostics to establish laboratory training workshops; and
assess availability of policies and guidelines related to management and control of diphtheria.

## Methods

A questionnaire used by the European Centre for Disease Prevention and Control (ECDC) to assess diphtheria diagnostic capacity (*6*) was adapted and sent to laboratories identified as part of the laboratory network for invasive bacterial diseases in the Western Pacific Region. Key topics covered in the survey included:

diptheria surveillance;
laboratory capacity and diagnostic services;
laboratory training, external quality assurance (EQA) and support needs;
serology and population immunity screening; and
public health (i.e. use of guidelines/manuals for diagnostics and case management, and availability of antitoxin).

Responses were validated by the Public Health  England (PHE) team. This included following up significant omissions or inconsistencies.

A set of criteria was defined against which diagnostic capacity could be evaluated and any gap identified. The criteria were adapted from those used by ECDC, which were originally developed based on the advice of a group of experts from PHE, ECDC and the WHO Regional Office for Europe. (*6*) The criteria assessed for minimum standards in three areas:

Area 1: Microbiological and epidemiological surveillance
Area 2: Laboratory diagnostic capacity
Area 3: Expertise in laboratory diagnostics.

The survey was sent to 18 laboratory contacts in 15 WHO Western Pacific Region countries, and responses were received from 17 contacts in 14 different countries. The Pacific island countries were assumed to have  limited diphtheria diagnostic capacity and the questionnaire was sent to Fiji only; however, there was no response from Fiji. The responses from two countries indicated that there were no laboratories capable of diphtheria diagnostic tests within their country. These same countries, however, did not return full survey responses and we were unable to infer the status of surveillance, policies and guidelines. These countries were excluded from the analysis due to missing/unknown information. They were therefore excluded from the analysis, but this already highlights a gap in diphtheria diagnostics within the Region.

In summary, responses were received from 17 laboratories (94%) in 14 countries (93%); however analysis was done for 15 laboratories (83%) in 12 countries (80%). The denominator for Area 1 was based on 12 countries, because this Area assessed the gap in micro/epi surveillance for which the survey responses were required. The denominator for Area 2 was based on 14 countries because this Area assessed the gap in laboratory capacity, for which there were limited responses from Cambodia and Papua New Guinea.

## Results

### Area 1: Microbiology and epidemiological surveillance

Gaps in microbiology and epidemiological surveillance were assessed against the following criteria:

Diphtheria should be a notifiable disease in every country.
Every country should have a surveillance system in place for diphtheria.
Every country should have close collaboration in place between microbiology and epidemiology for diphtheria surveillance.

All 12 countries reported diphtheria was a notifiable disease and had surveillance systems in place. Of these, 87% of laboratories reported a case-based surveillance system in their country (*n* = 13), and 13% reported aggregate surveillance (*n* = 2). One laboratory reported having a combination of case-based and aggregate surveillance. One country did not report a close collaboration between microbiology and epidemiology for surveillance. Overall, Area 1 of the gap analysis was met by 92% (*n* = 11) of countries.

### Area 2: Laboratory diagnostic capacity

Gaps in laboratory diagnostic capacity were assessed against the following criterion:

Each country should ideally have at least one laboratory at the reference laboratory level with additional expertise available through a regional reference laboratory and the WHO reference centre when required.

To reach reference laboratory standards, a laboratory must have at least one method for three analyses: microscopic examination (Gram stain or other), primary culture (blood agar or Tellurite agar) and biochemical identification and toxigenicity by either polymerase chain reaction (PCR) or modified Elek immunoprecipitation test.

Of the 14 surveyed countries, nine countries (64%) reported full reference-level capacity based on culture, biochemical identification and toxigenicity testing methods, and three countries (21%) reported partial diagnostic capacity. Two countries (14%) had no diphtheria laboratory diagnostic capacity at all.

Specific diagnostic issues identified include the  following:

Only six laboratories reported having capacity for molecular typing.
A range of tests were used for toxigenicity  testing; the majority of laboratories use PCR-based  methods (73%); six of laboratories (40%) use the Elek test.
Four out of 15 (27%) laboratories experienced problems in obtaining culture media for diphtheria diagnostics, and four reported issues with supplies of antitoxin for laboratory diagnostics.

### Area 3: Expertise in laboratory diagnostics

Gaps in expertise in laboratory diagnostics were assessed against the following criterion:

At least one current laboratory staff member should have received official training under the auspices of WHO on diphtheria identification and toxigenicity testing in the last five years.

No laboratory staff attended comprehensive external training workshops in the last five years, and 73% of contacts from 15 laboratories felt that a training workshop was needed.

### Other findings

None of the countries stated whether their surveillance encompassed *C. ulcerans* and *C. pseudotuberculosis* as well as *C. diphtheriae*. If surveillance is based on the WHO case definition, (*7*) then only *C.diphtheriae* is likely to be captured.
There is a lack of EQA for this specialized area of laboratory diagnostics.
Only four of 12 countries had the capability to undertake serological tests and had undertaken studies previously.
Nine laboratories (60%) across nine countries have diphtheria antitoxin procurement in place.

## Conclusions

### Key areas for action

The gap analysis demonstrated that there were gaps in diphtheria diagnostics within the WHO Western Pacific Region, with all responding countries fulfilling the minimum criteria for surveillance, specialized laboratory diagnostics and expertise. The areas with the greatest gaps are related to laboratory diagnostics expertise and surveillance of all three potentially toxigenic corynebacteria: *Corynebacterium diphtheriae*, *C.ulcerans* and *C.pseudotuberculosis*. Considering the adequate availability of funds for diphtheria, further studies are necessary. The following areas are highlighted as requiring further action:

Surveillance systems should ideally be in place for all three pathogens to detect and respond to diptheria; however, this is not mandatory at the moment as the WHO case definition only captures the disease diphtheria as caused by toxigenic strains of *C.diphtheriae*.
The laboratory diagnostic capability must be enhanced in some countries to isolate the causative pathogen, detect toxigenicity and undertake  molecular characterization of the above pathogens; hence, there is an urgent need for some countries’ laboratory staff to attend a laboratory training workshop for diphtheria diagnostics.
An EQA with participation from countries  attending the next training workshop needs to be established.
Adequate availability of specialized media and reagents for diphtheria diagnosis must be assured within the Region.
Updated guidelines for laboratory diagnosis of diphtheria should be made available.
Risks related to the lack of availability and procurement of DAT should be addressed.
